# A Diet Fortified with Anthocyanin-Rich Extract (RED) Reduces Ileal Inflammation in a Senescence-Prone Mice Model of Crohn’s-Disease-like Ileitis

**DOI:** 10.3390/antiox14040473

**Published:** 2025-04-15

**Authors:** Giulio Verna, Vicky Caponigro, Stefania De Santis, Emanuela Salviati, Fabrizio Merciai, Fabiano De Almeida Celio, Pietro Campiglia, Katia Petroni, Chiara Tonelli, Aurelia Scarano, Angelo Santino, Manuela Giovanna Basilicata, Marcello Chieppa, Fabio Cominelli

**Affiliations:** 1 Department of Medicine, Digestive Health Research Institute, Case Western Reserve University School of Medicine, Cleveland, OH 44106, USA; gxv71@case.edu (G.V.); fdc14@case.edu (F.D.A.C.); 2 Department of Pharmacy, School of Pharmacy, University of Salerno, 84084 Fisciano, Italy; vcaponigro@unisa.it (V.C.); esalviati@unisa.it (E.S.); fmerciai@unisa.it (F.M.); pcampiglia@unisa.it (P.C.); 3 Department of Pathology, Case Western Reserve University School of Medicine, Cleveland, OH 44106, USA; sxd951@case.edu; 4 Department of Biosciences, University of Milan, 20133 Milan, Italy; katia.petroni@unimi.it (K.P.); chiara.tonelli@unimi.it (C.T.); 5 Institute of Sciences of Food Production C.N.R., Unit of Lecce, 73100 Lecce, Italy; aurelia.scarano@cnr.it (A.S.); angelo.santino@cnr.it (A.S.); 6 Department of Advanced Medical and Surgical Sciences, University of Campania “Luigi Vanvitelli”, 80138 Naples, Italy; manuelagiovanna.basilicata@unicampania.it; 7 Department of Experimental Medicine (DIMeS), University of Salento, 73100 Lecce, Italy

**Keywords:** Crohn’s disease, polyphenols, adjuvant therapy, microbiota, metabolome

## Abstract

SAMP mice develop progressive Crohn’s disease (CD)-like ileitis without spontaneous colitis that worsens over time without chemical, genetic, or immunological manipulation. Even growing in an identical vivarium and fed with the same diet, SAMP mice reveal a distinct fecal microbiome, metabolome, and lipidome profile compared to AKR mice, their non-inflamed parental control strain. Differences are already present in 5-week-old mice, with a tendency to increase in 15-week-old mice. SAMP and AKR mice metabolome and lipidome profiles were substantially different, belonging to two clusters in line with the progression of intestinal disease. Similarly, the 16S analysis confirmed differences between 15-week-old AKR and SAMP mice. The protective role of dietary polyphenols has been documented in inflammatory bowel diseases (IBD); thus, we supplemented the chow diet with an anthocyanin-rich extract (RED) to evaluate disease reduction in SAMP mice and changes in fecal microbiota/metabolome. Our data reveal that 10-week supplementation with anthocyanin-rich extract ameliorated disease severity in SAMP mice despite limited fecal microbiota/metabolome differences.

## 1. Introduction

Inflammatory bowel diseases (IBDs) are gastrointestinal disorders driven by numerous factors, including genetic predisposition, dysregulated immune response, and strong dysbiosis, leading to chronic and relapsing diseases [1]. Apart from these factors, environmental ones support IBD pathogenesis; in fact, an unbalanced diet and limited bioactive nutritional compound intake may play a pivotal role in IBD onset and relapses, partially explaining the IBD incidence increase in Westernized countries [2]. Unbalanced and self-sustaining intestinal immune response is the signature of IBD, and several cytokines were associated with this condition; among these, tumor necrosis factor (TNF) was the first to be targeted by biological therapies based on monoclonal antibody infusion. Conventional therapies for IBD patients include corticosteroids and biological agents that have shown efficacy in maintenance therapy. Anti-TNF therapies (such as infliximab (IFX), adalimumab, and certolizumab) have revolutionized the treatment of CD in the past two decades. These agents have become increasingly widespread and commonly used [3,4,5]. Despite their wide use, approximately one-third of patients immediately fail to respond to anti-TNF drugs (primary non-responders), and 23–46% of patients lose response over time (secondary non-responders) [6,7]. Even in patients achieving complete remission, targeting inflammatory mediators may not be sufficient for long-term disease remission [8]. The persistence of environmental triggers may represent the reason for disease recurrence and/or a lack of response to biologicals. For example, intestinal dysbiosis may be involved in disease relapse, directly triggering inflammation or producing metabolites that may bend the immune response toward inflammation [9]. In light of the significant number of patients who are non-responders to the currently available therapies, it is crucial to define adjuvant therapies as able to act in parallel with biological drug administration.

Preclinical murine models of IBD have been intensively used to uncover several aspects of human pathology. These models include chemical induction, gene mutations, and cell transfer [10]. Each model has limits. Nonetheless, their use significantly increased the knowledge of disease onset and progression, shedding light on new targets for developing innovative therapies. Among in vivo models of ileitis, the SAMP1/YitFC (SAMP) mice have the unique feature of spontaneously developing early ileitis (starting at 10 weeks of age) without genetic or immunologic manipulations, which reaches 100% penetrance by 20 weeks of age, with progressively worse disease up to 60 weeks [11,12].

These mice’s typical macroscopic and histological features are focal areas of necrosis and inflammation in the ileum with a cobblestone pattern, crypt elongation, and immune cell infiltration. Immune phenotyping showed SAMP mice as a mixed Th1/Th2 model switching toward the Th2 type, mainly thanks to IL-33-driven pathways [11,13]. This model represents a valid murine counterpart of human disease, featuring similar macroscopic and microscopic features. For this reason, we used SAMP mice as a valuable tool to study the effects of anthocyanin-rich extract (RED) administration in in vivo models.

The microbiome and metabolome of SAMP mice have not been fully characterized to date. Thus, we employed 16S rRNA sequencing and UHPLC-MS/MS approaches to characterize the pre-inflamed and inflamed states of this experimental model of CD-like ileitis.

Several studies have already investigated the potential use of polyphenols and flavanols as primary or adjuvant therapies for IBD patients. Recent evidence highlights the role of polyphenols and their metabolites as modulators of the intestinal microbiota by shaping its composition and reducing chronic inflammation in the gut. Several pieces of evidence proved the benefits of polyphenol administration to human patients and murine models of chronic colitis [14,15]. Moreover, polyphenols exert a prebiotic-like activity by inhibiting pathogenic bacteria and promoting beneficial ones [16]. Polyphenol-enriched OGM, specifically bronze tomatoes, can reduce the inflammation in a preclinical model of UC, the Winnie mice, favoring the growth of beneficial bacteria [17]. The same can be said of the impact of bronze tomato on dendritic cells (DCs), where it decreased inflammatory cytokine secretion while increasing anti-inflammatory IL-10 production [18]. Among the suggested polyphenols’ mechanisms of action, their ability to act as natural iron chelators to sequestrate iron from both host and microbial species is fascinating. This peculiar characteristic is lost when immune cells are grown with an excess of iron [19].

A recent single-center study demonstrated that patients receiving a diet enriched with RED better reduced circulating inflammatory biomarkers compared to patients with a diet enriched with commercial red fruit tea (RFT), despite IFX being administered to both groups [20,21]. Importantly, we demonstrated that RED was more efficient in CD than in ulcerative colitis (UC) patients [20], although the metabo-lipidomics profiling revealed limited modulation induced by the flavonoid-rich diet vs. control [21]. Flavonoids are the most represented class of polyphenols, along with stilbenes, phenolic acids, and lignans, and can be further divided into flavan-3-ols, flavanols, flavones, isoflavones, flavanones, and anthocyanins. They are present in nature in glycoside and non-glycosylated form, and this can influence their bioavailability and metabolism. Polyphenols are primarily absorbed in the small intestine, but colonic bacteria can also metabolize them; in both cases, they can affect immune cells directly or indirectly [22]. Thus, our results sustain the potential role of polyphenols as bioactive phytochemicals, which can be used as adjuvant therapy in patients affected by chronic inflammatory disorders.

## 2. Materials and Methods

### 2.1. Animal Studies

Our investigations were performed under the relevant animal protocols, which were approved by the Institutional Animal Care and Use Committee (IACUC) protocol 2014-0158 at Case Western Reserve University (CWRU, Cleveland, OH, USA). All animals were maintained in a controlled environment (20–22 °C, 12 h light and 12 h dark cycles, and 45–55% relative humidity). We used the ARRIVE checklist for these studies. All animal protocols were approved by the Institutional Animal Care and Use Committee (protocol number 2014-0158). All animal studies were blinded without prior knowledge of the experimental groups by the experimenter. Mice were randomized using a progressive numerical order. The code for each mouse was known only to the animal caretaker and was revealed only at the end of the study. Single animals were used for all experiments, and an equal number of gender/age-matched animals were used in each group. Each animal experiment used a sample size of 6–12 mice and/or observations per group, which allowed for sufficient degrees of freedom to fit our statistical model. No criteria for inclusion or exclusion were set, and there was no exclusion of animals, experimental units, or data points. AKR mice were used as controls. AKR and SAMP colonies are maintained at the Animal Resource Center at Case Western Reserve University. All mice were maintained under specific pathogen-free conditions in ventilated micro-isolator cages with 1/8-inch corn bedding and cotton nestlets for environmental enrichment at the CWRU Animal Facility and kept on a 12 h light/dark cycle. Mice were routinely tested for specific pathogens. Mice had ad libitum access to water and were fed with a standard laboratory rodent diet P3000 (Harlan Teklad, Indianapolis, IN, USA). At the end of the treatment, the animals were euthanized by CO_2_ inhalation followed by cervical dislocation to confirm death. At the end of the treatment, colon tissues were harvested for histological assessment.

### 2.2. 16S rRNA Microbiome Analysis

Mouse stool was collected before (Day 0) and after the end of the RED treatment (Day 70). Following fecal DNA extraction using the QIAamp^®^ PowerFecal^®^ Pro DNA kit (QIAGEN, Hilden, Germany) according to the manufacturer’s protocol, microbiome amplification for the 16s rRNA gene V4 regions and high-throughput 16S rRNA gene microbiome sequencing and analysis were conducted using the well-established Illumina MiSeq and analytical protocols as previously described [17].

### 2.3. Metabolomics Sample Preparation and Analysis

A total of 200 mg of stool samples from the same animals used for the 16S sequencing were processed using the OMNImet^®^·GUT kit ME-200 (DNA Genotek, Ottawa, ON, Canada) before the metabolite extraction. The samples were then processed, as reported previously [23]. Following centrifugation, polar metabolites and lipid fractions were separately collected, dried by a SpeedVac (Savant, Thermo Scientific, Bremen, Germany), and stored until analysis. For the assessment of repeatability and instrument stability over time, a QC strategy was applied. Metabolomics and lipidomics were performed on two different analytical platforms, as reported previously [23,24].

### 2.4. Untargeted Lipidomics and Metabolomics

Lipid analysis was performed by RP-UHPLC-TIMS on a Thermo Ultimate RS 3000 coupled online to a TimsTOF Pro quadrupole Time of flight (Q-TOF) (Bruker Daltonics, Bremen, Germany). The separation was performed with an Acquity UPLC CSH TM C18 column (50 × 2.1 mm; 1.7 μm, 130 Å) protected with a VanGuard CSH TM precolumn (5.0 × 2.1 mm; 1.7 μm, 130 Å) (Waters, Milford, MA, USA). Untargeted metabolomics was carried out by HILIC-HRMS on a Thermo Ultimate RS 3000 coupled to a Q-Exactive quadrupole-Orbitrap (Thermo Scientific, Bremen, Germany). The separation was carried out with a Sequant ZIC-HILIC (100 × 2.1 mm; 3 μm) protected with a pre-column (5 × 21 mm; 3 μm) (Supelco, Bellefonte, PA, USA). Detailed parameters are reported elsewhere [23,25].

### 2.5. Statistical Analysis and Metabolomics Data Processing

Statistical analysis was performed using the GraphPad Prism 8 software (GraphPad Software, San Diego, CA, USA). All data obtained from at least three independent experiments were expressed as mean ± SEM. We evaluated statistical significance using the two-way ANOVA test following Sidak’s post hoc test. The results were considered statistically significant at *p* < 0.05.

Bioinformatics analyses of sequence data, from the processing of raw DNA sequences and reads to obtain alpha index estimates, were conducted in the QIIME2 microbiome platform (version 2020.8). Paired demultiplexed 16S sequences were denoised by using the q2-deblur QIIME plugin (https://github.com/qiime2/q2-deblur, accessed on 27 September 2022). Taxonomy was inferred by using the SILVA QIIME-compatible classifier (release 138). Alpha diversity metrics, including Shannon entropy and Faith’s PD, were also computed by using the QIIME2 platform [26,27]. Starting from QIIME2 relative abundances, the q2-emperor plugin was used to compute beta diversity metrics. Significant taxa among groups were computed by using a two-sided Welch test corrected by multiple tests with Benjamini–Hochberg.

Metabolomics and lipidomics HRMS data analyses were performed with MS-DIAL v4.48 (http://prime.psc.riken.jp/compms/msdial/main.html, accessed on 3 January 2024) and MetaboScape 2021 (Bruker), respectively. The detailed processing parameters for both metabolomics and lipidomics are reported elsewhere [28,29]. An enrichment and pathway analysis was performed with the relative tools in MetaboAnalyst 5.0 (https://www.metaboanalyst.ca/MetaboAnalyst/ModuleView.xhtml, accessed on 3 January 2024).

The omics data analysis was conducted using MATLAB R2024b (The MathWorks, Inc., Natick, MA, USA), combining built-in functions and custom scripts tailored specifically to the objectives of this research. The dataset comprised multiblock omics data derived from the same cohort of samples, encompassing metabolomics (ESI^+^ and ESI^−^) and lipidomics (ESI^+^ and ESI^−^). To ensure analytical rigor and robustness while minimizing bias and error, each dataset underwent individual preprocessing steps designed to enhance consistency and reliability for subsequent analyses. Data filtering was followed by normalization, wherein each variable was scaled relative to the median value of the corresponding sample to mitigate inter-sample variability. Missing or zero entries were addressed by substituting them with uniformly distributed random values, scaled by a factor equivalent to one-fifth of the smallest non-zero value within the respective column, thereby ensuring controlled and reproducible imputation.

Each omics block was then subjected to independent univariate statistical analysis using the Wilcoxon rank-sum test (Mann–Whitney U test) [30] to identify variables exhibiting significant differences between experimental groups. Multiple testing correction was performed using the False Discovery Rate (FDR) methodology to reduce the likelihood of type I errors [31]. Statistically significant variables were visualized through heatmaps, where the data are normalized by rows to enhance interpretability and emphasize relative differences within each variable across samples. These variables were further examined to identify trends indicative of underlying biochemical or physiological mechanisms. Post-normalization, a base-10 logarithmic transformation, was applied to the datasets to stabilize variance and minimize the influence of large-scale differences. External Parameter Orthogonalization (EPO) [32] was implemented to eliminate external biological variability, removing four principal components to preserve maximal informational content. The preprocessed data were then autoscaled, centering each variable to a mean of zero and scaling to unit variance, ensuring standardized comparisons across variables. Data fusion, also referred to as multiblock analysis, was employed as an integrative chemometric approach to simultaneously analyze multiple omics datasets [33]. This strategy facilitated the extraction of complementary information from distinct data blocks, enhancing the robustness, reliability, and predictive accuracy of the model. Exploratory analysis incorporated Common Component and Specific Weight Analysis (CCSWA), commonly termed ComDim, to identify both shared and unique sources of variation across the data blocks. This method iteratively identified global and local components that accounted for the maximum joint variability. The contribution of each data block to these components was quantified via the “Salience” metric. To ensure equitable representation, each data block was normalized to its Frobenius norm, standardizing the total variance among the blocks. The ComDim approach yielded score matrices that represent sample relationships derived from the simultaneous evaluation of all datasets [34,35]. Hotelling’s (T^2^) confidence ellipses were constructed for each experimental class and shown on the score plots, providing a 98% confidence level for visualizing sample distributions and class separations.

### 2.6. RED Diet Administration

SAMP and AKR mice were maintained under SPF conditions, fed with standard laboratory chow, and kept on 12 h light/dark cycles in the animal resource core (ARC) facility of CWRU. Five-week-old mice were randomly divided into two groups: vehicle vs. RED; the vehicle group received normal drinking water while the RED group received 53 mg/kg [36,37] of anthocyanin-rich powder obtained from purple corn [20] dissolved in their drinking water, changed each day for 70 consecutive days. Mice were checked every day and weighed every week. At day 70, all mice were euthanized according to the ARC protocols, and their distal ileum and colon were explanted, fixed in Bouin’s fixative (Sigma-Aldrich, St. Louis, MO, USA) for 24 h, and stored with 70% EtOH before paraffin embedding, sectioning, and staining for hematoxylin/eosin to be scored for inflammation by a trained pathologist. Colon length and weight were measured as indicators of colonic inflammation at sacrifice. The colon/body weight indices were expressed as a percentage calculated as the ratio of the colon length/weight and the total body weight (BW) for each mouse.

## 3. Results

### 3.1. Fecal Mice Microbiota in 5- and 15-Week-Old SAMP Mice

We performed a 16S rRNA sequencing on fecal material collected from AKR and SAMP mice at 5 and 15 weeks of age.

**Figure 1:**
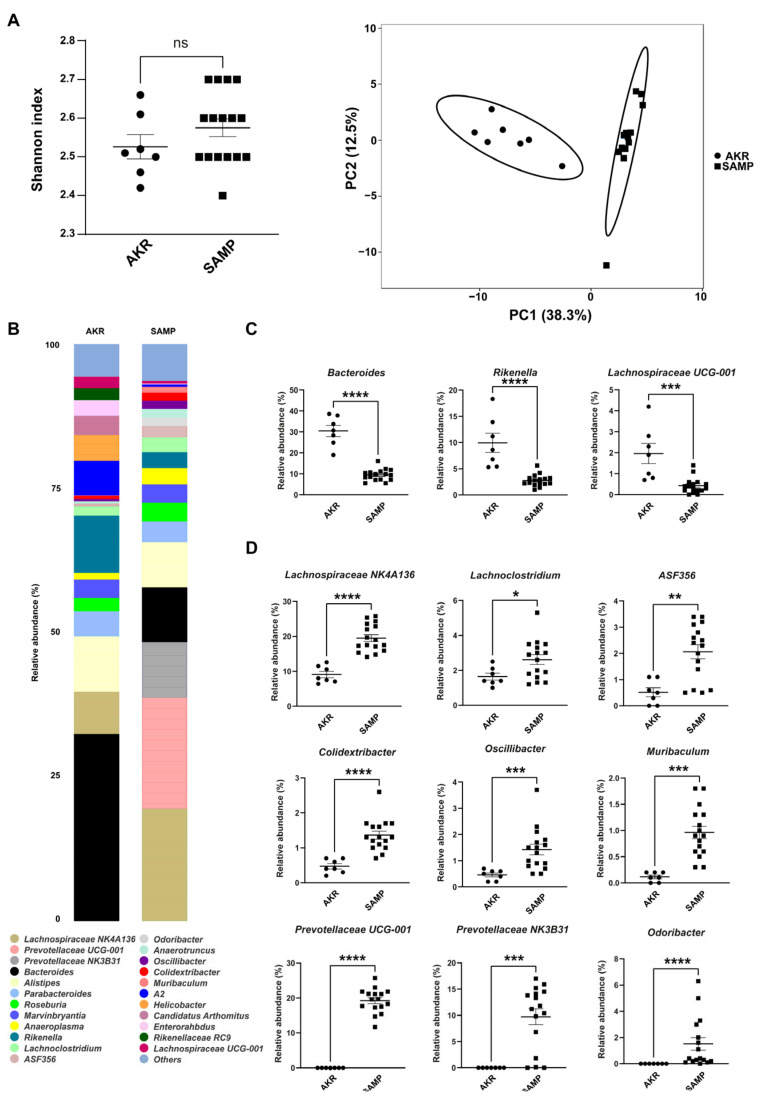
Shannon index and PCA plot for 5-week-old SAMP and AKR mice (**A**). Stacked bar plots for representative genera in AKR and SAMP mice (**B**). Dot plots for the significant genera found in 5-week-old AKR and SAMP mice (**C**,**D**) (* *p*-value < 0.05, ** *p*-value < 0.005, *** *p*-value < 0.001, and **** *p*-value < 0.0001). Data expressed as mean ± SEM (*n* = 16).

The data showed no differences in alpha diversity (Figure 1A, left panel), while the two genotypes separated well on the principal component analysis (PCA) plot (Figure 1A, right panel). The bar plots in Figure 1B show differences in the two cohorts of mice; 24 genera were the most abundant (above 1%) for each mouse strain, while several others were included in the “others” group (below 1%). Among the most abundant genera, 12 were significantly represented between AKR and SAMP mice (Figure 1C,D). *Bacteroides*, *Rikenella*, and *Lachnospiraceae UCG-001* were more represented in young 5-week-old SAMP mice compared to age-matched parental controls (AKR mice, Figure 1C). Conversely, nine genera were more abundant in young SAMP mice: *Lachnospiraceae NK4A136, Lachnoclostridium*, *ASF356*, *Colidextribacter*, *Oscillibacter, Muribaculum*, *Prevotellaceae UCG-001*, *Prevotellaceae NK3B31*, and *Odoribacter* (Figure 1D). Of note, in AKR mice, *Odoribacter*, *Prevotellaceae NK3B31*, and *Prevotellaceae UCG-001* were absent.

**Figure 2:**
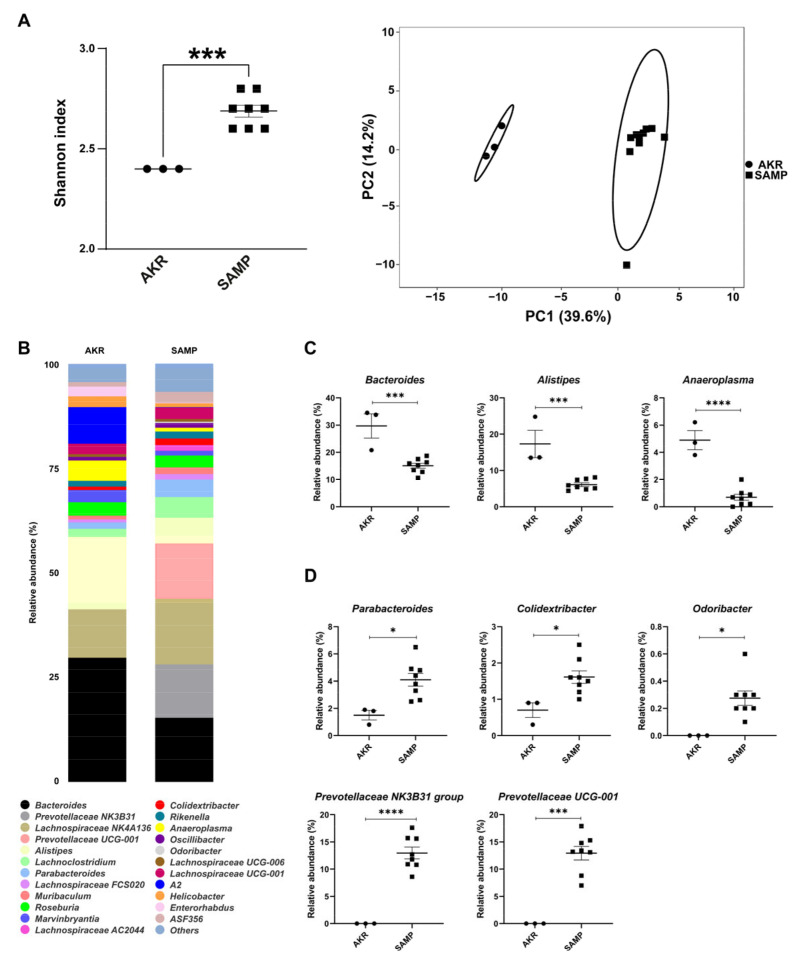
Shannon index and PCA plot for 15-week-old SAMP and AKR mice (**A**). Stacked bar plots for representative genera in the two strains (**B**). Dot plots for the significant genera found in 15-week-old SAMP and AKR mice (**C**,**D**) (* *p*-value < 0.05, *** *p*-value < 0.001, and **** *p*-value < 0.0001). Data expressed as mean ± SEM (*n* = 3 for AKR and 8 for SAMP mice).

The same analysis was repeated at 15 weeks in SAMP mice showing severe ileitis relative to AKR mice. Figure 2A shows an increased Shannon index between the two cohorts of mice and a great separation between them on the PCA plot. Figure 2B reports the 24 most abundant genera in AKR and SAMP mice. In particular, *Bacteroides*, *Alistipes*, and *Anaeroplasma* were found to be more abundant in AKR mice (Figure 2C), while *Parabacteroides*, *Colidextribacter*, *Odoribacter*, *Prevotellaceae NK3B31*, and *Prevotellaceae UCG-001* were more represented in SAMP mice (Figure 2D).

### 3.2. Fecal Mice Metabolome and Lipidome in 5-Week-Old AKR and SAMP Mice

**Figure 3:**
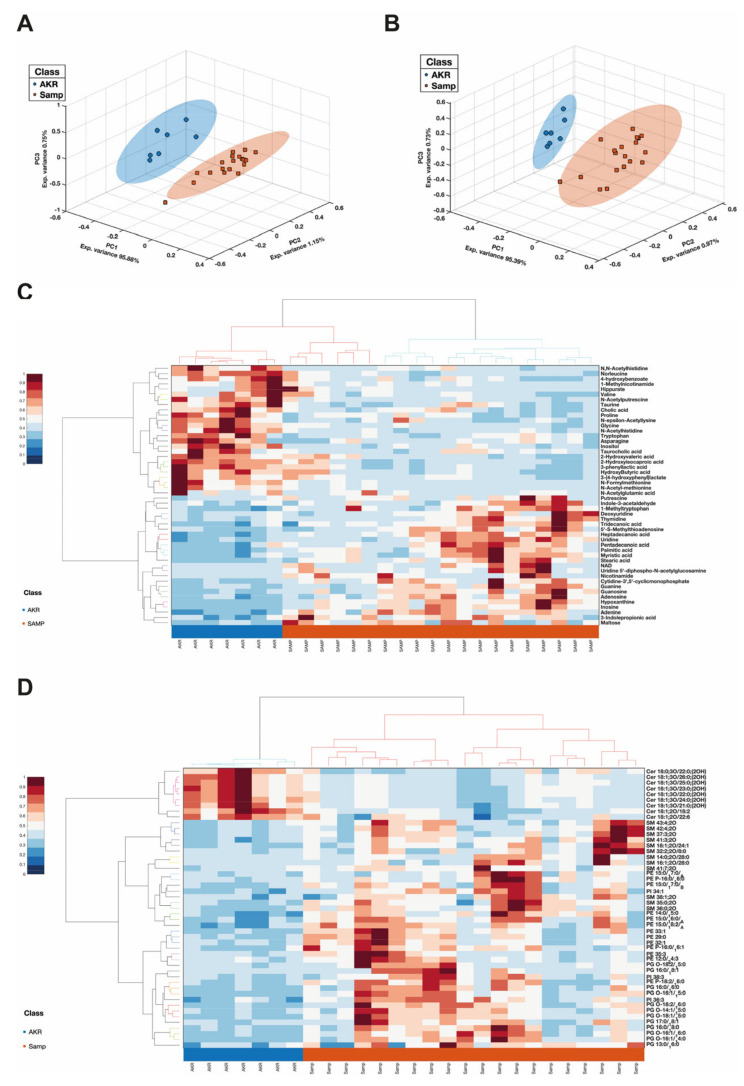
COMDIM score plots for the metabolome and lipidome of 5-week-old SAMP and AKR mice (**A**,**B**) and respective heatmaps showing the top 50 significantly modulated metabolites and lipids (*p*-value < 0.05) (**C**,**D**).

The metabolome and lipidome profiles of SAMP and AKR mice were substantially different at 5 weeks, as shown by PCA plots (Figure 3A,B). The loadings corresponding to each data block, along with their respective salience values, are provided in Supplementary Figure S1A,B, offering detailed insights into the contribution of individual blocks to the identified standard components. The heatmap in Figure 3C reports the top 50 differentially abundant metabolites. Among them, several were increased in SAMP mice and were mainly represented by purine and pyrimidine intermediates (adenine, cytidine, xanthine, uridine, adenosine, guanine, deoxyuridine, guanosine, hypoxanthine, and inosine), long-chain saturated fatty acids (LCFA with a carbon length from C13 to C18), tryptophan metabolites (e.g., indole-3-acetaldehyde, indole-3-propionic acid, and 1-methyltriptophan), polyamine metabolites (e.g., putrescine and 5′-S-methylthioadenosine), and nicotinamide metabolism intermediates (e.g., NAD and nicotinamide). On the contrary, many others were reduced in SAMP compared to AKR mice. These were essentially amino acids and N-derivatives (acetyl, methyl, formyl, bile acids, and cholic and taurocholic acid), hydroxy fatty acids, taurine, and hippurate. Concerning lipids, as observed from the heatmap in Figure 3D, many lipids were found to be increased in SAMP vs. AKR mice. They were mainly represented by phosphatidylethanolamines (PEs), phosphatidylglycerols (PGs), phosphatidylinositol (PI), and sphingomyelins (SMs). On the other hand, the lipids found to be reduced in the same comparison were primarily represented by Ceramides (CERs), diacylglycerols (DGs), and lysophosphatidylcholines (LPCs).

**Figure 4:**
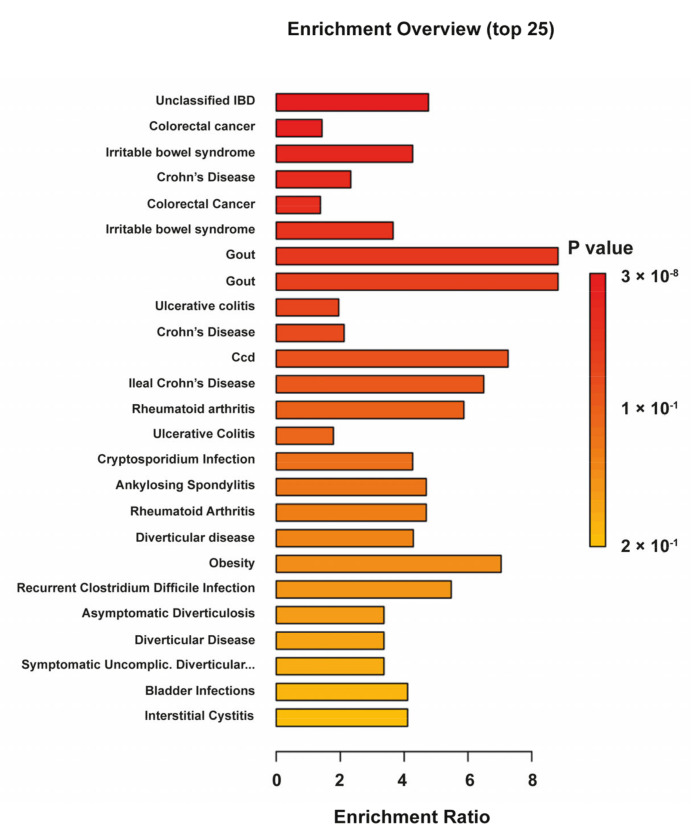
Quantitative enrichment analysis (QEA) overview presenting the top 25 related metabolic pathways ranked according to the fold enrichment *p*-value.

The human metabolome database (HMDB) codes were used to build a KEGG enrichment analysis with stools used as the biospecimen background. Figure 4 shows the top-enriched pathways, including unclassified IBD, UC, and CD, thus highlighting the presence of key metabolites and lipids for the two forms of the disease.

### 3.3. In Vivo Effects of RED Administration in SAMP Mice

Given the importance of microbiota in the onset of chronic colitis and the efficacy of polyphenols in modulating microbial populations in both human patients and mouse models, we recently proved that RED extract enhanced the IFX response in CD patients [20,21]. To test the role of RED in preventing intestinal inflammation in susceptible individuals, we administered this extract to SAMP mice without clear signs of inflammation. Specifically, 5-week-old mice were administered with RED powder (53 mg/kg) dissolved in sterile water or a vehicle (control group) for 70 days; AKR mice were used as parental controls for each condition.

**Figure 5:**
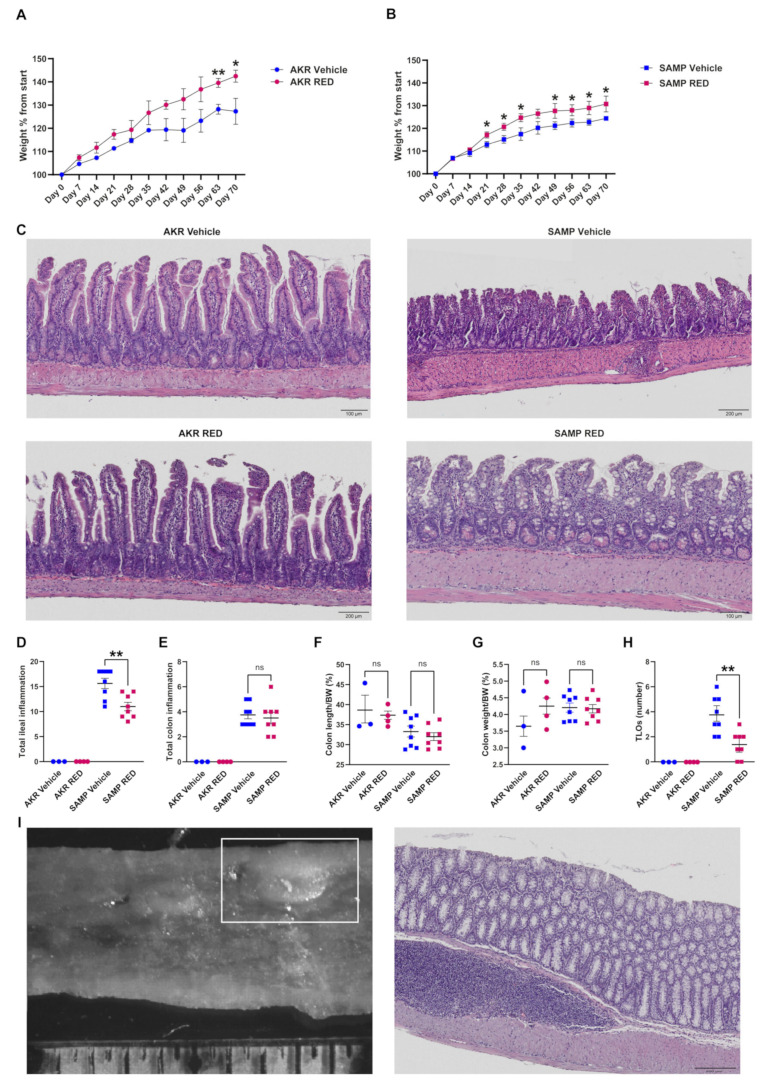
In vivo treatment of SAMP and AKR mice with RED. Five-week-old SAMP mice were administered with RED powder (53 mg/kg) or vehicle for 70 days; AKR mice were used as parental controls for each condition. Weight was measured each week for all the treatment (10 weeks) in AKR (**A**) and SAMP (**B**) mice. Representative H&E-stained ilea from AKR (left panel) and SAMP (right panel) mice treated with vehicle (top row) or RED (top row) (**C**). Inflammatory score of ileal inflammation (**D**) and colon inflammation (**E**) was calculated for all the experimental groups, as well as colon length/BW (**F**) and colon weight/BW (**G**) ratio percentage measured at the time of sacrifice, and TLOs count (**H**). Representative TLO stereomicroscopy of RED-treated SAMP mice and its respective H&E (**I**). Magnification: 10×. Abbreviation: BW: body weight. (* *p*-value < 0.05 and ** *p*-value < 0.005). Data expressed as mean ± SEM (*n =* 3 for AKR vehicle, 4 SAMP vehicle, 8 AKR RED, and 8 SAMP RED).

The mice’s weight was recorded every week for 10 weeks. RED-treated AKR mice gained more weight than the vehicle-treated group, but the average weight was significantly different only after 9 and 10 weeks of RED administration (Figure 5A). Starting from the third week, RED administration induced SAMP mice weight gain that remained significantly higher than vehicle-treated SAMP mice up to the end of treatment (Figure 5B).

In line with this, histology scores from the ileum highlighted a significant inflammation reduction in RED-treated compared to vehicle-treated SAMP mice (Figure 5C,D). In particular, H&E-stained ilea from RED-treated SAMP mice showed a reduction in the inflammatory *milieu* with less immune cell infiltration and necrotic areas and a more conserved villi architecture than the vehicle-treated ones (Figure 5C). The AKR mice did not show any sign of inflammation or morphologic differences between the two groups (Figure 5C,D). A relevant result induced by RED administration was the reduction in tertiary lymphoid organs (TLOs), mainly in the distal tract of SAMP mice, despite the absence of other differences in histological score and macroscopic parameters (Figure 5E–H). The results are also highlighted by the stereomicroscopy and its relative H&E staining (Figure 5I, left and right, respectively, and Supplementary Figure S4).

Supplementary Figure S1A,B shows the score plots of a COMDIM analysis for the metabolome and lipidome of the SAMP and AKR mice after the RED treatment (both the control and treated groups). The marked separation between the SAMP and AKR mice is still present; however, the RED treatment does not appear to induce appreciable clustering between the experimental groups before and after the RED treatment, as shown in Supplementary Figure S2A,B. These data are confirmed in Supplementary Figure S3, showing the details for metabolomics (Supplementary Figure S3A,B) and lipidomics (Supplementary Figure S3C,D) in 15-week-old SAMP and AKR mice before and after the RED treatment.

Supplementary Figure S4 shows representative stereomicroscopies of vehicle- and RED-treated SAMP mice with the detailed inset of the inflamed colon mucosa and the TLO.

**Figure 6:**
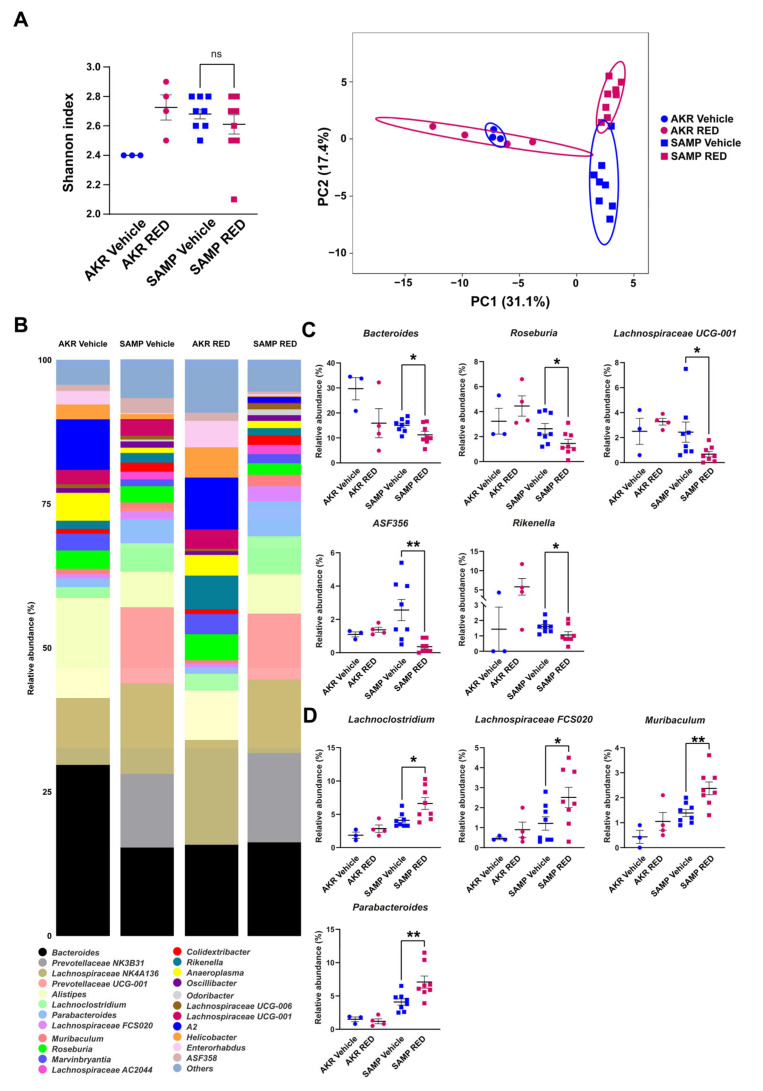
Shannon index and PCA plot for 15-week-old AKR and SAMP mice after RED treatment (**A**). Stacked bar plots for representative genera in the two strains (**B**). Dot plots for the significant genera modulated by RED treatment in 15-week-old SAMP and AKR mice (**C**,**D**) (* *p*-value < 0.05 and ** *p*-value < 0.005). Data expressed as mean ± SEM (*n* = 3 for AKR vehicle, 4 SAMP vehicle, 8 AKR RED, and 8 SAMP RED).

The alpha diversity of the fecal microbiota of the 15-week-old SAMP mice did not change in response to 10 weeks of RED administration, and vice versa, AKR mice showed a trend to increase their alpha diversity in the RED-treated group, reaching the same level as the SAMP group (Figure 6A); although some genera were different, with the present numerosity, both groups did not reach significance following RED. PCA shows no separation between the two AKR groups, while there is little distancing of RED-treated SAMP mice from the vehicle-treated ones. The bar plots in Figure 6B show all the top abundant genera in our experimental groups before the sacrifice. The 16S analysis revealed a reduction in the genera of *Bacteroides*, *Roseburia*, *Lachnospiraceae UCG-001*, *ASF356*, and *Rikenella* and an increase in the *Lachnoclostridium*, *Lachnospiraceae FCS020*, *Muribaculum*, and *Parabacteroides* genera in the RED-treated SAMP mice (Figure 6C). No differences were observed in the AKR mice based on the RED treatment.

## 4. Discussion

Standard therapies used to reduce inflammation in IBD patients are effective in most cases, but some patients fail to respond to them over time. Thus, there is an urgent need to create new adjuvant methods to boost anti-inflammatory drug potential further. Polyphenols can cover this niche as they have often been proven effective immunomodulators in vitro and in vivo. RED administration to IBD patients reduced serum inflammatory cytokine concentrations and positively modulated their stool metabolome and microbiota [21].

Microbiota has a central role in the onset and pathogenesis of chronic colitis. Thus, we wanted to study stool microbiota and metabolites of the SAMP model over time, before and after the onset of inflammation. Stools collected right after the weaning of SAMP and AKR mice resulted in 11 microbial genera differentially represented. Among them, *ASF356* (part of the *Clostridiales* family) was more expressed in the SAMP mice, and it can induce a Th17 response [38] that is associated with CD in both human and mouse models [39]. The same trend was observed with *Colidextribacter*, a genus that is present in the biofilm of the inflamed colon [40]; it is also present in the early days after DSS administration [41], and it is also associated with gut barrier dysfunction in human patients [42]. *Lachnoclostridium* was also associated with inflammation and increased in stool from CD patients [43,44]. *Lachnospiraceae NK4A136*’s association with inflammation is debated, but it was found to be related to gastritis and DSS colitis as a mucin-consumer genus [45,46]. Nevertheless, this genus’s abundance was lower and not statistically different from the AKR group at later time points, indicating that it might have some beneficial effects [47] that are lost with increased inflammation in older mice. *Muribaculum*’s role in IBD is unclear, and little evidence shows that its abundance is reduced after the onset of inflammation [48]. *Oscillibacter* was found to be related to inactive CD in patients, mirroring a state of predisposition to the onset of disease we observe in SAMP mice [49]. Lastly, both *Prevotellaceae* genera are absent in AKR mice, indicating their exclusive part of the SAMP mice microbiota [50].

On the other hand, *Rikenella* showed some beneficial and protective effects against intestinal inflammation, and its lower abundance in young SAMP mice might boost the odds of developing ileitis [51]. *Lachnospiraceae UCG-001* are responsible for SCFA production [52], and we found them more abundant in young AKR mice. Altogether, these more abundant genera in AKR mice have protective effects; thus, their absence in SAMP mice might be related to the progressive onset of intestinal inflammation. Despite the increased abundance of *Bacteroides* in AKR mice, a genus with notorious pathogenic strains, some species still have protective and homeostatic effects on gut health; thus, a better analysis is needed to clarify their role in AKR mice’s gut.

Stool metabolomics and lipidomics exhibited a profound difference between the SAMP and AKR mice immediately after weaning. Among the differentially expressed metabolites, purine and pyrimidine intermediates were highly abundant in the SAMP mice feces. The alteration in purine homeostasis is tightly linked with gut bacterial metabolism, and increased purine and pyrimidine levels have been associated with increased demand, such as in colon cancer [53] or the increased turnover of epithelial cells [54], as well as playing a role in exacerbating colitis [55]. Nevertheless, their levels should be correlated with tissue or plasma concentrations. LCFA levels were increased in the SAMP mice. LCFA has an ambiguous role in UC. In some observations, saturated LCFA has been associated with IBD induction and aggravation [56]. Mouse models in which 2,4,6-trinitrobenzene sulfonic acid (TNBS) was used to induce colitis also showed greater LCFA end products than healthy controls with the same diet [57].

Additionally, saturated LCFA has been associated with dysbiosis and a specific bacterial composition [58]. Some polyamine intermediates, such as putrescine and SAM, were increased in the SAMP mice, and these have been ascribed to the disruption of the epithelial tight junction in ex vivo and in vivo models [59]. Interestingly, this last aspect is counterbalanced by taurine, which decreased compared to the AKR group associated with intestinal epithelial integrity. The role of bile acid metabolism is still debated in IBD models; some observations reported that some cholic acids have anti-inflammatory effects and can also be used for the treatment of inflammation-related diseases, including colitis [60]. Reduced bile acid levels of cholic and taurocholic acid characterized the SAMP group. In addition, the levels of multiple hydroxy fatty acids were found to increase, among them beta-hydroxybutyric acid, which has been recently identified to promote macrophage polarization and reduce the severity of acute experimental colitis [61]. Among different lipid subclasses, the SAMP group was characterized by an overall reduction in Ceramides (CERs) and increased sphingomyelins (SMs) fecal content. A decrease in Ceramide synthesis has been associated with disrupted barrier function [62,63]. The consequent increase in SM could suggest an imbalance of acid sphingomyelinase (aSMase), an essential sphingomyelin hydrolase. Of note, a profound glycerophospholipid remodeling was observed in SAMP mice, with phosphatidylethanolamine (PE), phosphatidylglycerols (PGs), and phosphatidylinositols (PIs). Interestingly, a substantial number of PG species were found to increase, and these have been associated with gut dysbiosis and inflammation [64].

In 15-week-old mice, *Colidextribacter* and *Prevotellaceae* abundance was unchanged between the SAMP and AKR mice. *Bacteroides* increased in the SAMP mice, albeit they are still less abundant than in the AKR mice. We observed an increased abundance of *Parabacteroides* and *Odoribacter* in the SAMP mice; both genera characterize the microbiota of ileitis-affected mice [48]. Moreover, *Anaeroplasma*, which we found more abundant in the AKR mice, is related to IgA and TGFβ production by immune cells in murine Peyer’s patches [65,66]. Despite the old SAMP mice showing a higher abundance of *Alistipes* [50], we found a higher abundance of this genus in the AKR mice, probably because it might increase its abundance with aging. Thus, the inflammatory conditions of the SAMP mice may not be supported by *Alistipes* outgrowth.

To validate the protective effects of RED anthocyanins in a CD model, we administered RED powder extract to the SAMP and AKR mice starting from 5-week-old mice for the following 70 days. Weekly weight monitoring showed increased weight gain in both mouse groups that received RED. Weight loss is often considered among the symptoms of colitis, and a positive weight gain can be regarded as an improvement in mice’s health, even though a weight increase was also observed in the control group.

RED anti-inflammatory effects were confirmed by SAMP mice’s lower ileal inflammatory score after 70 days of treatment, from 5 to 15 weeks of age. Compared to vehicle-treated SAMP mice, we observed healthier villi and less immune cell infiltration in the ilea of mice that received RED in their beverage. Moreover, RED reduced the formation of TLOs [67] in the colon of SAMP mice; these immune organs are responsible for sustaining chronic inflammation and are regarded as a hallmark of the CD phenotype in mice [68].

Their reduction can be seen as an improvement in the intestinal tract’s general inflammatory status, even though a lower number of TLOs can increase DSS susceptibility [69].

This change in both groups might be attributable to the increase in beneficial bacteria that grew more abundant during the RED treatment, such as *Lachnospiraceae*, *Lachnoclostridium*, and the less abundant *Bacteroides*. *Lachnospiraceae* are often responsible for IBD pathogenesis and are increased in CD patients, which can be influenced by polyphenols [70,71]. *Parabacteroides* were also found to be upregulated after the administration of polyphenols, and their role in the gut is often regarded as protective and anti-inflammatory [72,73]. The *Lachnoclostridum* genus was found abundantly in blueberry-treated rats, indicating that anthocyanin-rich foods might increase their presence and role in intestinal homeostasis [74]. At the same time, it was also seen to increase in mice treated with probiotics and amylase, which is associated with SCFA production [75]. *Rikenella* and *Roseburia* genera are related to IBD and inflammation, and there is evidence of anthocyanin modulation in their presence [51,76]; *Roseburia* was also inhibited by *Sonchus arvensis* extracts and had positive effects against DSS-induced colitis [77]. Lastly, the *Bacteroides* genus was found to have a predominant role in SAMP ileitis pathogenesis [78] and is often associated with an inflamed gut. This change in the microbiota has positive effects on the production of SCFA, as we also observed in the metabolomic analysis. *Lachnoclostridium* is a genus of succinate-transforming bacteria. Succinate, moreover, is responsible for mucosal healing and protection in *Tnf*^ΔARE/+^ mice [79].

*Parabacteroides* are linked to the production of succinate and linoleic acid, as well as their metabolites [80]. There is evidence of anti-inflammatory effects [81], an immunomodulatory capability on macrophages [82], and metabolism changes mediated by them [83,84]. *ASF356*’s decreased abundance could be related to polyphenols’ protective effects on microbiota composition [85], while *Muribaculum*’s increased abundance [86,87] was also observed in inflammation models treated with plant polyphenols.

Metabolomes and lipidomes are substantially different between AKR and SAMP mice at both 5 and 15 weeks of age. RED administration did not induce consistent variation in metabolome and lipidome clusters in both genotypes, suggesting that, at least in these experimental conditions, the RED-protective effects are mainly directed to the host tissues and not mediated by altered metabolic production by the intestinal flora.

Several plant extracts, similar to RED in our model, help restore gut microbial balance by promoting beneficial bacteria, inhibiting pathogens, reducing inflammation, and modulating the gut barrier. Epigallocatechin-3-gallate (EGCG), the widely used extract from green tea, favors the growth of *Bifidobacterium* and *Lactobacillus* and limits the growth of *Clostridium* [88]. Totum-070, a combination of olive leaves, artichoke leaves, chrysanthellum, goji fruits, and black pepper, increased the *Muribaculum* and *Parabacteroides* genera in Westernized diet-fed mice [89]. Berberine is an extract obtained from the roots of plants common in traditional Chinese/East Asian medicines, including *Coptis chinensis*, *Berberis aristata,* etc. The administration of berberine in murine models of IBD enriched the relative abundance of *Firmicutes* and decreased *Proteobacteria* [90]. Positive effects on the *Bacteroides* genus were also obtained in a DSS model treated with cannabigerol hemp extract and black rice anthocyanins [91,92]. Several other plant extracts showed positive effects in murine models of spontaneous or chemically induced colitis, highlighting nutraceuticals’ potential to reshape intestinal microbiota and act as an adjuvant in disease remission [93,94,95].

Despite the reduced inflammation in the colon of the RED-treated SAMP mice, the selected low dose of anthocyanin was neither sufficient to induce a complete remission nor managed to affect the ileitis in these mice. Furthermore, this low dosage mildly reshaped the intestinal microbiota of the SAMP and AKR mice without a major influence on stool metabolites. We know that microbial metabolites and postbiotics might be the new frontiers of IBD therapy, so further research needs to be carried out to better determine anthocyanin effects on intestinal microbiota and metabolomics.

## 5. Conclusions

RED proved useful in reducing inflammation in the SAMP model of CD. RED administration was sufficient to change the SAMP intestinal microbiota despite the stool metabolome analysis not revealing a consistent shift toward a more eubiotic microenvironment. The current results demonstrate that RED administration might be useful as an adjuvant for the prevention/amelioration of CD onset, tackling luminal aspects of CD. Future studies will address the effects of combined treatments of RED with biological agents to evaluate if this strategy may support disease remission and reduce disease relapse.

## Data Availability

The raw data supporting the conclusions of this article will be made available by the authors upon request.

## Supplementary Materials

The following supporting information can be downloaded at https://www.mdpi.com/article/10.3390/antiox14040473/s1: Figure S1: COMDIM plots and loading plots for the metabolomics (A) and lipidomics (B) of 5-week-old AKR and SAMP mice; Figure S2: Cumulative COMDIM plots and loading plots for the metabolomics (A) and lipidomics (B) of all the mice at the different time points and experimental conditions; Figure S3: COMDIM plots and loading plots detailing the metabolomics of vehicle- and RED-treated SAMP mice (A) and AKR mice (B) and lipidomics of vehicle- and RED-treated SAMP mice (C) and AKR mice (D). Figure S4: Representative stereomicroscopies of vehicle- and RED-treated SAMP mice with the respective H&E zoom-in inset.

## Abbreviations

The following abbreviations are used in this manuscript:

RED	Anthocyanin-rich extract
CD	Crohn’s disease
SAMP	Senescence-prone mice
IBD	Inflammatory bowel disease
TNF	Tumor necrosis factor
IFX	Infliximab
TH1	T-helper cell 1
TH2	T-helper cell 2
IL-33	Interleukin 33
DCs	Dendritic cells
UC	Ulcerative colitis
RFT	Red-fruit tea
IACUC	Institutional Animal Care and Use Committee
SPF	Specific pathogen-free
ARC	Animal research center
CWRU	Case Western Reserve University
FDR	False Discovery Rate
EPO	External Parameter Orthogonalization
CCSWA	Common Component and Specific Weight Analysis
PCA	Principal component analysis
DSS	Dextran-sodium sulfate
LCFA	Long-chain fatty acids
SCFA	Short-chain fatty acids
TNBS	2,4,6-Trinitrobenzenesulfonic acid
CERs	Ceramides
SM	Sphingomyelin
PE	Phosphatidylethanolamine
PGs	Phosphatidylglycerols
PI	Phosphatidylinositol
LPCs	Lysophosphatidylcholines
HDMB	Human metabolome database
BW	Body weight
TLOs	Tertiary lymphoid organs
