# Supplementary figures and images for: A Diet Fortified with Anthocyanin-Rich Extract (RED) Reduces Ileal Inflammation in a Senescence-Prone Mice Model of Crohn’s-Disease-like Ileitis
Source: Antioxidants (Basel). 2025 Apr 15;14(4):473. doi: 10.3390/antiox14040473 (PMC12024068; doi:10.3390/antiox14040473)

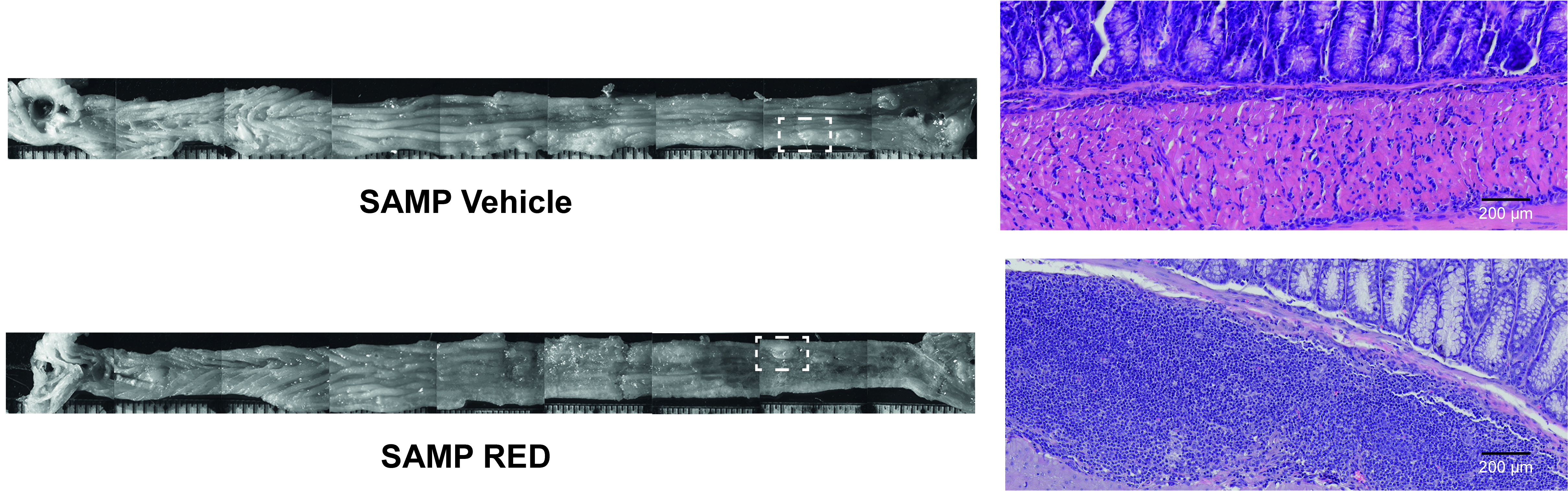

Supplement: Supplementary file 1 [file antioxidants-14-00473-s001.zip › Figure S4.tif]
